# Functional Variability of Snake Venom Metalloproteinases: Adaptive Advantages in Targeting Different Prey and Implications for Human Envenomation

**DOI:** 10.1371/journal.pone.0109651

**Published:** 2014-10-14

**Authors:** Juliana L. Bernardoni, Leijiane F. Sousa, Luciana S. Wermelinger, Aline S. Lopes, Benedito C. Prezoto, Solange M. T. Serrano, Russolina B. Zingali, Ana M. Moura-da-Silva

**Affiliations:** 1 Laboratório de Imunopatologia, Instituto Butantan, São Paulo, SP, Brazil; 2 Laboratório de Hemostasia e Venenos, Instituto de Bioquímica Médica, Universidade Federal do Rio de Janeiro, Rio do Janeiro, RJ, Brazil; 3 Laboratório Especial de Toxinologia Aplicada, Instituto Butantan, São Paulo, SP, Brazil; 4 Laboratório de Farmacologia, Instituto Butantan, São Paulo, SP, Brazil; 5 Laboratório de Fisiopatologia da Trombose, Faculdade de Farmácia, Universidade Federal do Rio de Janeiro, Rio do Janeiro, RJ, Brazil; 6 Center of Toxins, Immune-Response and Cell Signaling (CeTICS), FAPESP, São Paulo, SP, Brazil; Russian Academy of Sciences, Institute for Biological Instrumentation, Russian Federation

## Abstract

Snake venom metalloproteinases (SVMPs) are major components in most viperid venoms that induce disturbances in the hemostatic system and tissues of animals envenomated by snakes. These disturbances are involved in human pathology of snake bites and appear to be essential for the capture and digestion of snake's prey and avoidance of predators. SVMPs are a versatile family of venom toxins acting on different hemostatic targets which are present in venoms in distinct structural forms. However, the reason why a large number of different SVMPs are expressed in some venoms is still unclear. In this study, we evaluated the interference of five isolated SVMPs in blood coagulation of humans, birds and small rodents. P-III class SVMPs (fractions Ic, IIb and IIc) possess gelatinolytic and hemorrhagic activities, and, of these, two also show fibrinolytic activity. P-I class SVMPs (fractions IVa and IVb) are only fibrinolytic. P-III class SVMPs reduced clotting time of human plasma. Fraction IIc was characterized as prothrombin activator and fraction Ic as factor X activator. In the absence of Ca^2+^, a firm clot was observed in chicken blood samples with fractions Ic, IIb and partially with fraction IIc. In contrast, without Ca^2+^, only fraction IIc was able to induce a firm clot in rat blood. In conclusion, functionally distinct forms of SVMPs were found in *B. neuwiedi* venom that affect distinct mechanisms in the coagulation system of humans, birds and small rodents. Distinct SVMPs appear to be more specialized to rat or chicken blood, strengthening the current hypothesis that toxin diversity enhances the possibilities of the snakes for hunting different prey or evading different predators. This functional diversity also impacts the complexity of human envenoming since different hemostatic mechanisms will be targeted by SVMPs accounting for the complexity of the response of humans to venoms.

## Introduction

Venoms are trophic adaptations which allow venomous snakes to use potent toxins as a chemical means to subdue their prey [1]. This efficient predation mechanism evolved in venomous snakes by recruitment of genes coding for physiological functions to the venom system in which they underwent duplications followed by neofunctionalization of the new copies under accelerated evolution [2]–[5]. Under these mechanisms of evolution, gene copies that encode venom components usually conserve non-synonymous mutations and result in protein sequences with high degree of variability. It has also becoming evident that gene expression patterns of the different copies may also be variable representing an additional source of diversity in the final venom composition that may be responsible for inter- or intraspecific differences in toxins [6].

In snakes, it is reasonable to assume that the major protein families recruited to the venom apparatus have been selected by their efficiency to subdue prey [7]. In this regard, venoms of elapid snakes and from certain viperid species are very efficient in prey paralysis and killing due to potent neurotoxins enclosed within three-finger toxins and phospholipases A_2_ (PLA_2_) protein families. The presence of proteolytic enzymes, especially snake venom metalloproteinases (SVMPs), is also noted in snake venoms and some authors suggest that they may be involved in predigestion of prey [8]. However, in viperid venoms, the three-finger proteins are rare and PLA_2_s are not abundant while metalloproteinases, particularly P-III class SVMPs (for classification of SVMPs see Fox & Serrano [9]), acquire an apparent evolutionary advantage since they are expressed in high levels in the venomes of most species of vipers [7].

The expression of SVMP or PLA_2_ phenotypes has been recently discussed by Mackessy [10] in venoms of North American *Crotalus spp*. The authors classified *Crotalus* venoms as Type I (predominantly SVMPs) or Type II (predominantly PLA_2_s). Amongst several examples, there was an inverse correlation in the amount of PLA_2_ compared to SVMP depending on geographical distribution of Mojave rattlesnakes [11]. In *Crotalus spp*, phospholipase activity of type II venoms presented a correlation with neurotoxicity and lethality [10], but the exact function of SVMPs in type I venoms is poorly understood. This is particularly important in the case of *Bothrops* snakes in which PLA_2_ molecules lost their neurotoxic activity and instead present a tissue-damaging myotoxic activity [12]. Perhaps, as a consequence, most species of *Bothrops* have type I venoms with high contents of SVMPs and it is plausible that these enzymes have acquired important functions for subduing prey or even evading predators in these snakes. In this aspect, the potent action of proteinases on hemostatic system has been correlated to prey immobilization and killing, besides their assumed digestive function [13]. Indeed, SVMPs, particularly from P-III class, are related to the hydrolysis of extracellular matrix components [14]–[16], coagulation factors [17], inhibition of platelet aggregation [18], [19], apoptosis of endothelial cells [20], [21] and up-regulation of pro-inflammatory mediators expression [22], [23] leading to a massive disruption of hemostasis that results in hemorrhages and severe tissue lesions at the venom inoculation site [24]. These mechanisms enhance the action of snake venom serine proteinases, present in lower levels in both type I and type II venoms, that are thrombin-like enzymes with procoagulant, anticoagulant, platelet aggregating and fibrinolytic activities [25].

Besides the importance of venom variability for snake ecology, this topic also has relevance for a correct understanding of venom composition for treatment of snake bites. For example, in the case of the Mojave rattlesnake, human envenomings by type II venom specimens were more severe than by type I [11] due to their neurotoxic activity. In *Bothrops* snakes the envenomation scenario is the opposite. The classical symptoms of envenoming by *Bothrops spp* are hemostatic disturbances and inflammatory reaction with tissue damage extending from the site of the bite [26] mainly due to the SVMP effects. Thus, the variability among components of SVMP protein family could be implied in the complexity of the mechanisms of action leading to the local tissue damaging. Therefore, knowing the correlation between different structural homologs and their function is essential to guarantee the production of antivenoms able to neutralize toxins acting on different targets.

Although the polarization between type I and type II venoms is currently accepted, it has becoming evident that some venoms occupy intermediate state between types I and II, with a wider range of different toxins, indicating that their major toxin pattern might still be under selection. Therefore, these venoms serve as important models to understand functional variability of venom components and the major killing strategies of individual toxins. The proteome of *Bothrops neuwiedi* venom has been recently characterized by our group [27] as an intermediate composition between type I and type II venoms. In this analysis, SVMPs accounted for almost 50% of venom proteome, however, with predominance of P-I class SVMPs. In addition, PLA_2_s were present in a considerable amount of almost 10% [27]. Similarly, the venome of *B. pauloensis* (initially classified as *B. neuwiedi pauloensis*) also showed an intermediate type of composition in which 38% consisted of SVMPs (26% of P-III class SVMPs) and 32% of PLA_2_s [28]. Another interesting aspect of *B. neuwiedi* venom is that a substantial structural variability was detected within SVMPs. Analyzing the cDNA sequences obtained from the venom gland of one *B. neuwiedi* specimen, we described the transcription of at least eleven different SVMP sequences with distinct domain structures and variable degrees of domain identity [29]. These data confirmed the existence of a wide variety of SVMP toxins in *B. neuwiedi* venom, some not yet characterized, with structural differences that might be relevant from the point of view of venom functional variability. Working with different snake populations, Dagda and coworkers [30] demonstrated the genetic basis for intraspecies variation in metalloproteinase-associated functions of Mojave rattlesnake venom. The authors described four distinct genes coding for SVMPs and their level of expression in different individuals explained the potencies of caseinolytic, fibrinolytic, and hemorrhagic activities in the whole venom samples [30].

The diversity of SVMPs in the venoms highlight the importance of this class of toxins for the adaptation of *Bothrops* snakes and brings out the question of why venoms should contain such a number of distinct forms of toxins from the same functional/enzymatic group, presumably with similar activities. In this work we approached this question using several experimental models and our results strongly suggest that the variability between members of the same toxin family present adaptive advantages for the snake. The characterization of SVMPs isolated from *B. neuwiedi* venom showed a marked diversity against targets of mammalian hemostatic system and remarkable differences in their effects on rodent or bird blood coagulation systems, suggesting the presence of effective arsenals against different potential prey or even predators.

## Material and Methods

### Venom

A pool of venoms from snakes of *B. neuwiedi* complex was extracted from adult specimens of both sexes kept under captivity at Instituto Butantan herpetarium. This includes the subspecies *B. n. pauloensis, B. n. matogrossensis, B. n. neuwiedi, B. n. marmoratus* and *B. n. diporus* [31]. After extraction, the venom was lyophilized and kept frozen until use.

### Animals

Adult female *Gallus domesticus (white leghorn* chickens - 1.0 to 1.7 kg, 10 to 15 weeks) were purchased from commercial source. Male *Rattus norvegicus* (Wistar rats - 250 to 300 g) and *Mus musculus* (Swiss mice - 18 to 20 g) were bred at the Instituto Butantan. The animals had free access to water and food, and were kept under a 12-h light/dark cycle.

### Ethical statement

All experiments involving mice, rats and chickens were approved by the Ethical Committee for Animal Research of the Instituto Butantan (CEUAIB), São Paulo, Brazil, identification number 1028/13, who certified its agreement with the Ethical Principles in Animal Research adopted by the Brazilian Council of Animal Experimentation Control (CONCEA).

### 2-D Gel Electrophoresis

For two-dimensional gel electrophoresis, 150 µg of the venom proteins were dissolved in Milli-Q water and mixed with DeStreak rehydration solution containing 1% IPG (immobilized pH gradient) buffer (GE Healthcare, Uppsala, Sweden) to a final volume of 125 µL. The mixture was homogenized at room temperature and remained at rest for 30 min before centrifugation for 5 min at 12000 *g* and the supernatant was loaded on a 7 cm Immobiline DryStrip (pH 3–10, nonlinear, GE Healthcare) for equilibration for 18 h. First dimension IEF was carried out in an Ettan IPGphor Isoeletric Focusing System (GE Healthcare) at 20°C using a three-phase program: 500 V at 10 V/h; 4000 V at 3400 V/h; and 5000 V at 4600 V/h. After first dimension separation, the strip was placed in the rehydration tray and the proteins in the strip were washed, reduced and alkylated by sequential 20 min incubation steps in the following solutions: a) 50 mM Tris-HCl, pH 8.4, 2% (m/v) SDS (sodium dodecyl sulfate), 30% (v/v) glycerol, and 6 M urea (equilibration buffer, EB); b) 20 mg/mL dithiothreitol (DTT) in EB; and c) 30 mg/mL iodocetamide in EB. Then, the strip was directly applied to 12.5% polyacrylamide gel (10 cm×10 cm×1.0 mm) and submitted to electrophoresis at 170 V for the second dimension separation. The gels were stained with Coomassie blue or transferred to PVDF membranes. The blotted membranes were immersed in a blocking solution [5% non-fat milk in Tris-saline (20 mM Tris–HCl, 0.15 M NaCl, 0.05% v/v Tween 20, pH 7.4)] and then incubated with 1∶1,000 diluted anti-class P-III SVMP (jararhagin) antibodies [32]. After washing with Tris-saline, the membranes were incubated with peroxidase labeled rabbit anti-horse IgG (1∶1,000) and reactive bands detected by incubation with 4-chlor-α-naphtol and H_2_O_2_. The figures shown represent two independent runs.

### Venom fractionation

Venom was fractionated by molecular exclusion and ion exchange chromatographies using the Äkta system (GE Healthcare). Samples of 50 mg crude lyophilized venom were dissolved in 200 µL of 20 mM Tris, 1 mM CaCl_2_ and 150 mM NaCl, pH 7.8, and insoluble material was removed by centrifugation at 10,000 g for 10 min at room temperature. Proteins in the supernatant were applied to a Hiprep 16/60 S-200 column (GE Healthcare) in the same buffer and subjected to isocratic elution at a flow rate of 0.5 mL/min. The fractions containing metalloproteinase activities (hemorrhagic and fibrinolytic) were pooled according to major peaks and dialyzed against 20 mM Tris, 1 mM CaCl_2_, pH 7.8 and applied to a Mono-Q HR 5/5 column (GE Healthcare) using the same buffer. Proteins from size exclusion peaks 1 and 2 were eluted by a 40 mL gradient from 0 to 0.25 M NaCl and 5 mL gradient from 0.25 to 1 M NaCl, at a flow rate of 1 mL/min in the same buffer. Proteins of size exclusion peak 4 were eluted by a 20 mL gradient from 0 to 0.4 M NaCl and an extra 5 mL gradient from 0.4 to 1 M NaCl, at a flow rate of 1 mL/min in the same buffer. All steps were carried out at room temperature and monitored at 280 nm. Purity of toxins was evaluated by SDS-PAGE 12.5%, under non-reducing conditions. Samples containing 10 µg of protein were dissolved in sample buffer (0.125 M Tris-HCl, pH 6.8 containing 10% glycerol, 2% SDS, and 0.001% bromophenol blue), boiled for 5 min and applied to the gel. Electrophoresis was performed at a current of 35 mA until the bromophenol blue indicator reaches the end of the gel. The gels were Coomassie or silver stained using standard protocols. Molecular weight markers: phosphorylase b (97 kDa), bovine serum albumin (66 kDa), ovalbumin (45 kDa), bovine carbonic anhydrase (30 kDa), trypsin inhibitor (20.1 kDa), and lactalbumin (14.4 kDa).

### Protein identification by mass spectrometry

Protein bands of interest were excised from polyacrylamide gels and submitted to in gel digestion using trypsin according to Hanna *et al.* (2000). After the digestion procedure, the samples were reconstituted in 15 µL of 0.1% (v/v) formic acid and an aliquot (4.5 µL) of the resulting peptide mixture was injected on nanoAcquity UPLC Symmet C18 precolunm (180 µm i.d.×20 mm) (Waters, Milford, MA) and fractionated on a nanoAcquity UPLC BEH130 C18 column (75 µm i.d.×100 mm) (Waters, Milford, MA) at 35°C using a UPLC-ESI-Q-TOF system (Waters, Milford, MA, USA). Chromatography was performed with solvent A (0.1% formic acid in deionized water) and B (0.1% formic acid in acetonitrile) at a flow rate of 600 nL/min using the following gradient: 7% B in 1 min; to 45% B in 15 min; to 80% B in 2.5 min; hold at 80% B for 1 min; then back to 7% B in 1.5 min. The MS instrument was operated in data dependent mode, in which one full MS scan was acquired in the *m/z* range of 200–2000 Da followed by MS/MS acquisition using collision induced dissociation. The resulting fragment spectra were searched using MASCOT search engine (Matrix Science, UK) using serpentes (25210 sequences) database download from the Uniprot website (http://www.uniprot.org/) with a parent and fragment tolerance of 1.2 and 0.6 Da, respectively. Iodoacetamide derivative of cysteine and oxidation of methionine were specified in MASCOT as fixed and variable modifications, respectively.

### Hemorrhagic activity

Hemorrhagic activity was assessed using samples of 10 µg of proteins diluted in 50 µL PBS, and injected *i.d.* into the dorsal skin of Swiss mice. Control mice received only 50 µL PBS. After 3 h, the animals were sacrificed by CO_2_ inhalation, the dorsal skin removed and the extent of hemorrhagic lesion was calculated as the longest diameter multiplied by the diameter perpendicular to it. Results are expressed as mean ±SD of three mice in each group.

### Fibrinolytic activity

Fibrinolytic activity was assayed by the fibrin-plate method, as previously described [33]. Briefly, a fibrin-agarose gel was prepared by mixing 3 mg/mL human fibrinogen (Calbiochem) with a pre-heated solution of 2% agarose in 50 mM Tris–HCl buffer pH 7.3 containing 200 mM NaCl, 50 mM CaCl_2_ and 1 U/mL thrombin. Samples (10 µg) were applied to wells of the solidified gel and incubated at 37°C for 18 h, and then the area of the hydrolysis halo was measured and expressed as mean ±SD of three independent experiments.

### Recalcification time

Samples (50 µL) of isolated SVMPs (0.1; 0.5; 1; 5 e 10 µg/mL) or TBS (used as negative control) were incubated for 1 minute with platelet poor plasma (PPP 50 µL). The reaction was initiated by addition of 100 µL of 0.025 M CaCl_2_, and the clotting time (in seconds) was measured in a Coagulometer (KC 4 Delta - Tcoag version: v2.35). Results are expressed as mean ±SD of three independent experiments.

### Thrombin and FXa generation assay

Samples (50 µL) of isolated SVMPs (1 µg/mL) diluted in Hepes-BSA buffer pH 7.5 (50 mM Hepes, 100 mM NaCl, 5 mM CaCl_2_ and 1 mg/mL BSA) were coated in microplates. Reaction was started by addition of 50 µL/well prothrombin or factor X (2 µM and 0,4 µM, respectively) and aliquots of 10 µL were removed after incubation for 1, 5, 10, 15 and 20 min at 37°C and loaded into microplate wells containing 40 µL of stop buffer (50 mM Tris, 150 mM NaCl, 0.1% PEG 6000 and 20 mM EDTA, pH 7.5). After the addition of 50 µL of 200 µM S-2238 (D-Phe-Pip-Arg-pNA) or S-2765 D-Arg-Gly-Arg-pNA) (), the absorbance at 405 nm was recorded at 37°C for 20 min at 9s intervals using a Thermomax Microplate Reader (Molecular Devices). Thrombin and factor Xa were used as control samples. Results are expressed as mean ±SD of three independent experiments.

### Collection of citrated rat and chicken whole blood samples

Male Wistar rats (250 to 300 g) were anesthetized with sodium pentobarbital (60 mg kg^-1^; intraperitoneally). Blood samples (3 mL) drawn from the carotid arteries were collected into syringes containing 1∶10 (v/v) 3.8% trisodium citrate. Chickens were restrained on their backs with wings spread. Feathers were removed and, after using xylocaine spray as a local anesthetic agent, small incisions were made around the brachial wing vein and blood samples (5 mL) carefully collected into syringes containing 1∶10 (v/v) 3.8% trisodium citrate.

### Thromboelastometric assays

Thrombelastometric assays using the ROTEM four-channel system (Pentapharm) were performed according to the manufacturer's instructions. Volumes (300 µL) of citrated chicken or rat whole blood samples were preloaded with 40 µL containing: Group 1 – only PBS (control, n = 3); Group 2–10 µg of crude venom (n = 3) and Group 3–10 µg of isolated SVMPs (n = 3). The final mixture of 340 µL was placed into the ROTEM cups and incubated up to 60 min for evaluating the following parameters of the ROTEM profile: clotting time (CT [s], the time from the start of the assay until formation of a clot with an amplitude of 2 mm - green); clot formation time (CFT [s], time from the end of CT [amplitude of 2 mm] until a clot firmness of 20 mm is achieved - pink); maximum clot firmness (MCF [mm], the peak strength of the clot, resulting from the interaction of fibrin, activated platelets and factor XIII [FXIII] - blue); and maximum lysis (ML [%], the maximum reduction in clot firmness observed after MCF has been reached, given as a percentage of the MCF value).

## Results

**Figure 1:**
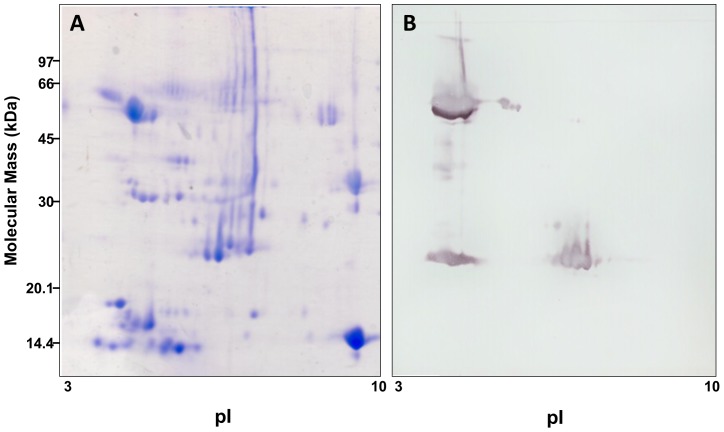
Characterization of different SVMPs *Bothrops neuwiedi* venom. Samples (150 µg) of *B. neuwiedi* venom were analyzed by 2-D gel electrophoresis in the pH range 3–10 and stained by Coomassie blue (A) or electrotransferred to nitrocellulose membranes and revealed with anti-jararhagin antibody followed by peroxidase labeled anti-rabbit IgG and enzyme substrate (B).

The structural diversity of SVMPs was first confirmed in a pool of venoms collected from *B. neuwiedi* snakes kept under captivity at the herpetarium of Instituto Butantan. When subjected to 2-D electrophoresis, the venom was fractionated in several spots distributed between p*I* 3 to 10 and apparent molecular masses between 10 kDa and 97 kDa (Figure 1A). Proteins transferred to PVDF membranes were then treated with antibodies against a P-III class SVMP (jararhagin), and the resulting molecular masses of the stained spots corresponded to the two major classes of SVMPs: a major group of around 60 kDa and acidic p*I*, compatible to the P-III class, and two spots of approximately 25 kDa revealed in acidic and neutral regions of the gel, which are characteristic of the P-I class (Figure 1B). These results confirm the presence of different SVMPs in *B. neuwiedi* venom.

**Figure 2:**
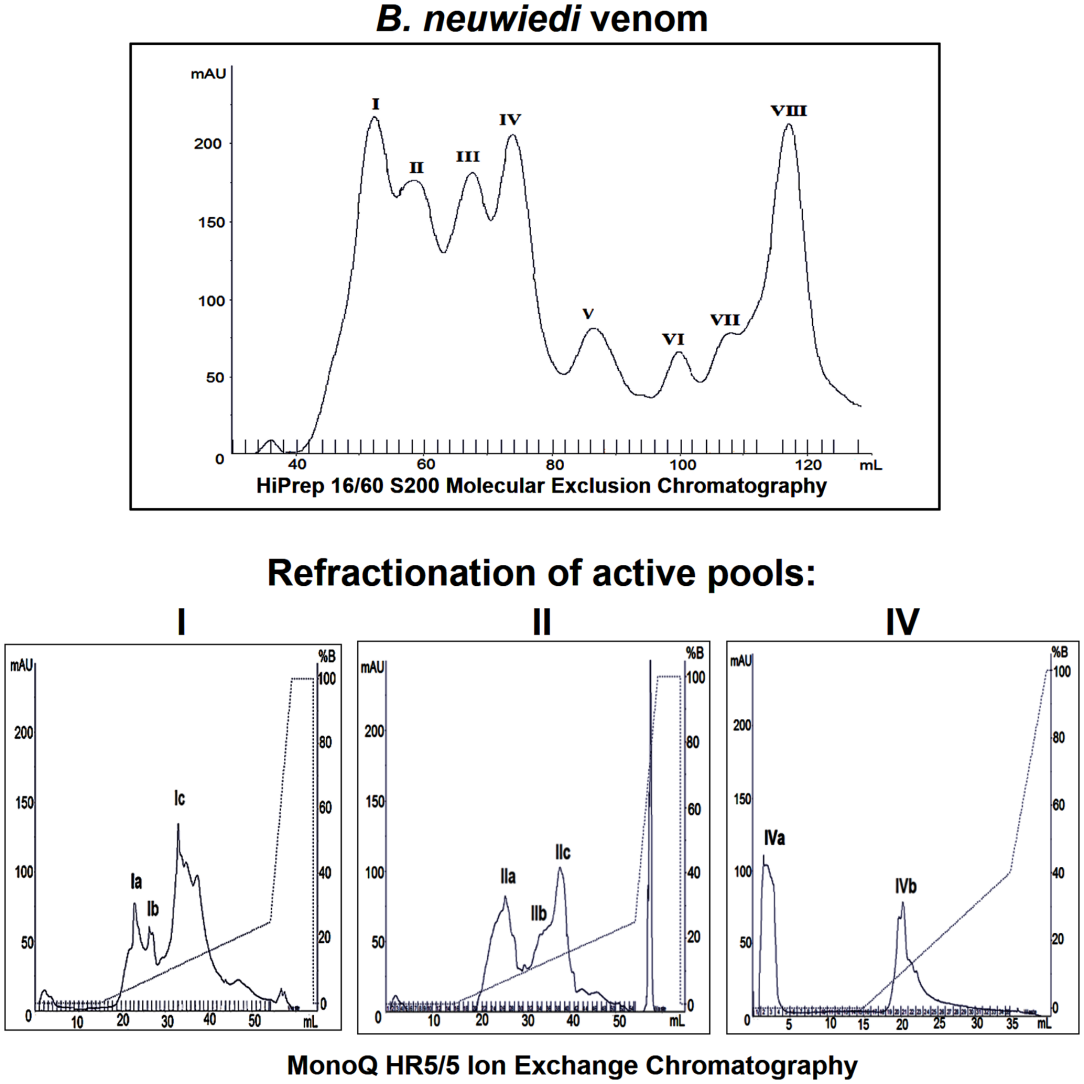
Isolation of *Bothrops neuwiedi* venom fractions enriched in SVMPs. (A) Samples of 50 mg of *B. neuwiedi* venom were applied on Hiprep 16/60 S200 column, equilibrated with 20 mM Tris, 150 mM NaCl and 1 mM CaCl_2_ pH 7.8. Fractions of 2.0 mL were collected at a flow rate of 0.5 mL/min, monitored at A280 (nm). (B,C and D) Fractions I, II and IV of size exclusion chromatography were applied to a Mono-Q HR 5/5, equilibrated with 20 mM Tris pH 7.8 plus 1 mM CaCl2. Fractions of 1.0 mL were collected at a flow rate of 1.0 mL/min, monitored at A280 (nm).

**Figure 3:**
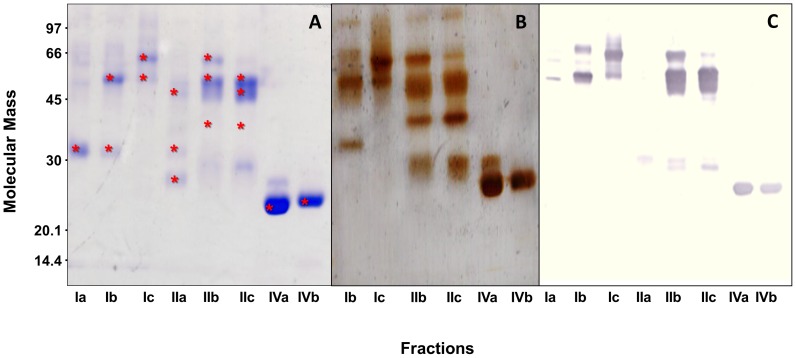
SDS-PAGE and Western blotting of the isolated fractions. Isolated fractions enriched with SVMPs (10 µg) were subjected to SDS-PAGE under non-reducing conditions and stained by Coomassie blue (A), silver stained (B) or electrotransferred to nitrocellulose membranes and revealed with anti-jararhagin antibody followed by peroxidase labeled anti-rabbit IgG and enzyme substrate (C). The bands with asterisks were cut and analyzed by mass spectrometry.

To assess the functional diversity of the distinct structural forms, *B. neuwiedi* venom was subjected to a series of preparative chromatographies and fractions enriched with the SVMPs that were obtained. Size exclusion chromatography resolved *B. neuwiedi* venom in eight major fractions enumerated I to VIII (Figure 2A). The SVMPs main activities (hemorrhagic, fibrinolytic and reactivity to anti-SVMP antibodies) were concentrated in the fractions I, II and IV (data not shown) that were selected for the next anion-exchange chromatographic step. When submitted to anion-exchange chromatography at pH 7.8, fraction I was resolved into three peaks denominated Ia, Ib and Ic (Figure 2B) and peaks Ib and Ic showed hemorrhagic activity and were selected for further analysis. Fraction II was also resolved into three peaks denominated IIa, IIb and IIc (Figure 2C) and two of them (IIb and IIc) were selected since they presented hemorrhagic and fibrinolytic activities. Fraction IV was resolved into two peaks denominated IVa and IVb (Figure 2D) with fibrinolytic activity that were also selected. All selected fractions reacted with anti-jararhagin antibodies, confirming their immunological identity with SVMPs (Figure 3C). Figure 3A shows the SDS-PAGE profile of selected fractions. Except for fractions Ia and IIa, the fractions contained major bands of molecular masses between 50–60 kDa and around 25 kDa, corresponding to SVMPs of the P-III and P-I classes, respectively. The major bands of each fraction were cut off the gel and subjected to mass spectrometry for protein identification. The fractions containing bands with SVMP molecular masses were run in another gel subjected to silver staining (Figure 3B).

As shown in Table 1, the fraction Ia major band showed only peptides characteristic of serine proteinases and fraction IIa showed three bands, two of them characterized as serine proteinases, therefore, these fractions were not selected for the functional tests. Fraction Ib showed two major bands, the band of 34 kDa was characterized as a serine proteinase and the 57 kDa band was identified as a *B. neuwiedi* SVMP class P-III (BnMPIIIb). However, due to the contamination with serine proteinases, this fraction was also rejected for the next step. Fraction Ic presented two bands, and the band of 67 kDa was identified as a *Bothrops neuwiedi* SVMP class P-III (BnMPIIIc). The three bands of fraction IIb included peptides of SVMPs class P-III (BnMPIIIc, bothropasin and jararhagin) and the three bands of fraction IIc were identified as bothropasin or jararhagin, indicating that the bands correspond to similar SVMPs class P-III probably at different processing stages due to autolysis of these toxins generating 30 kDa fragments corresponding to the disintegrin-cysteine-rich domains, as previously reported [32]. The bands of fractions IVa and IVb showed molecular masses characteristic SVMPs of class P-I and were identified as *B. neuwiedi* SVMPs (BnMPIb and BnMPIa, respectively), which are very similar in their protein sequence but differ in the isoeletric point and binding to the anion exchange columns [34].

**Table 1:** Assignment of the proteins isolated from the SDS-PAGE bands of B. neuwiedi venom fractions to protein families by MS/MS and MASCOT.

Fractions	M. Mass	Peptides	Identified Proteins	Species	*gi* number	Score
		DXMXXR				
**Ia**	**34 kDa**	FXAFXYPGR	BthaT1	*B. alternatus*	**giΙ48093528Ι**	**375**
		SVPNDDEEXR	serine proteinase			
		VXGGDECDXNEHR				
		NNGNLDEIR				
		NPCCDATTCK				
**Ib**	**57 kDa**	ENVIITPCAQEDVK	BnMPIIIb - SVMP	*B. neuwiedi*	**giΙ308212498Ι**	**427**
		KENVIITPCAQEDVK				
		SECDIAESCTGQSAQCPTDDFHK				
	**34 kDa**	SVPNDDEEXR	BthaT1 - serine proteinase	*B. alternatus*	**giΙ48093528Ι**	**120**
		NNGNLDEIR				
**Ic**	**67 kDa**	IALVGIEIWSNR	BnMPIIIc - SVMP	*B. neuwiedi*	**giΙ308212500Ι**	**218**
		IYEIVNFLNEIFR				
	**57 kDa**	-	No Hit			
	**53 kDa**	TXCAGVXBGGK	Serine proteinase	*B. insularis*	**giΙ20069139Ι**	**124**
		SVANDDEVXR				
**IIa**	**34 kDa**	SVPNDDEEXR	BthTa1 - serine proteinase	*B. alternatus*	**giΙ48093528Ι**	**69**
		MYEXANXVNEXXR*				
	**27 kDa**	NNGDXDKXK	Bothropasin-SVMP	*B. jararaca*	**giΙ4895110Ι**	**373**
		MYEXANXVNEXFR**	Jararhagin-SVMP		**giΙ62468Ι**	**166**
		XTVKPDVDYTXNSFAEWR				
		NNGNLDEIR				
		GDEYFYCR				
		YPCCDAATCK				
	**67 kDa**	IALVGIEIWSNR	BnMPIIIc - SVMP	*B. neuwiedi*	**giΙ308212500Ι**	**792**
		IYEIVNFLNEIFR				
		YINVYKPQCILNEPLR				
		SECDIAESCTGQSAECPIDDFKR				
		NNGDXDKXK				
**IIb**		XPCAPEDVK	Bothropasin - SVMP		**giΙ4895110Ι**	**551**
		KXPCAPEDVK				
	**57 kDa**	DNSPGBNNPCK		*B. jararaca*		
		MYEXANXVNEXXR*				
		MYEXANXVNEXFR**	Jararhagin - SVMP		**giΙ62468Ι**	**298**
		XTVKPDVDYTXNSFAEWR				
		XPCAPEDVK				
	**51 kDa**	KXPCAPEDVK	Jararhagin - SVMP	*B. jararaca*	**giΙ62468Ι**	**110**
		DNSPGBNNPCK				
		NNGDXDKXK				
		XPCAPEDVK				
	**57 kDa**	KXPCAPEDVK	Bothropasin - SVMP	*B. jararaca*	**giΙ4895110Ι**	**561**
		DNSPGBNNPCK				
		MYELANXVNEXXR				
		XTVKPDVDYTXNSFAEWR				
		NNGDXDKXK				
		XPCAPEDVK	Bothropasin - SVMP		**giΙ4895110Ι**	**508**
		KXPCAPEDVK				
	**53 kDa**	DNSPGBNNPCK		*B. jararaca*		
**IIc**		MYEXANXVNEXXR*	Jararhagin - SVMP		**giΙ62468Ι**	**353**
		MYEXANXVNEXFR**				
		XTVKPDVDYTXNSFAEWR				
		ASMSECDPAEHCTGBSSECPADVFHK				
		XPCAPEDVK				
	**51 kDa**	KXPCAPEDVK	Jararhagin - SVMP	*B. jararaca*	**giΙ62468Ι**	**173**
		DNSPGBNNPCK				
		ASMSECDPAEHCTGBSSECPADVFHK				
		DLIKVEK				
		YNSNVNTIR				
		TLTSFGEWR				
**IVa**	**22 kDa**	SCIMASTISK	BnMPIb - SVMP	*B. neuwiedi*	**giΙ308212504Ι**	**1956**
		YIELAVVADHGMFTK				
		TWVHEMVNSLNGFFR				
		SMNVDASLVNLEVWSK				
		VAVTMAHELGHNLGMDHDDTCTCGAK				
		DLIKVEK				
		ERDLLPR				
		KDLIKVEK				
		YNSNLNTIR				
**IVb**	**23 kDa**	TLTSFGEWR	BnMPIa - SVMP	*B. neuwiedi*	**giΙ308212516Ι**	**1820**
		VHEMVNTVNGFFR				
		YVELAVVADNGMFTK				
		SMNVDASLANLEVWSK				
		SMNVDASLANLEVWSKK				
		ISHDHAQLLTTIVFDNYVIGITK				

X =  Leucine or Isoleucine, B =  Lysine or Glutamine,

*Found only in Bothropasin sequence,

**Found only in Jararhagin sequence. C- or N-terminal peptides are underlined.

**Figure 4:**
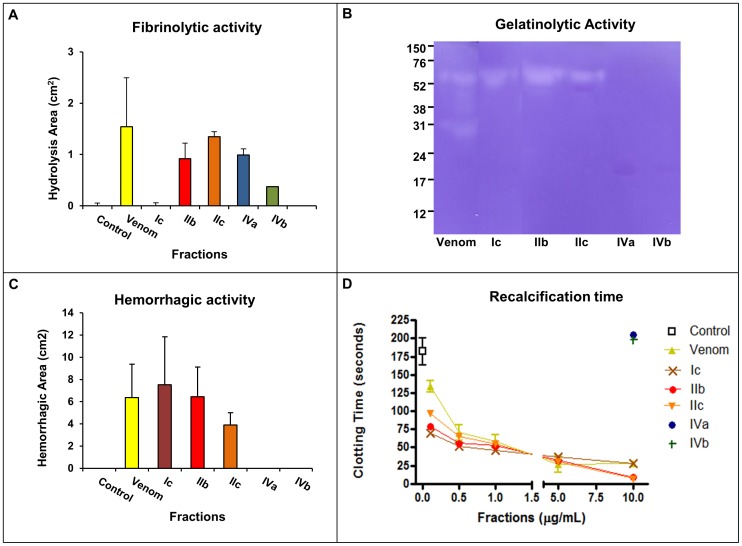
Fibrinolytic, Gelatinolytic, Hemorrhagic activity and Recalcification time of SVMPs. (A) Samples of 10 µg of each fraction was applied to the fibrin-agarose plate and the halos of lysis were measured. (B) Gelatinolytic activity of SVMPs (5 µg) on a 12.5% polyacrylamide gel copolymerized with gelatin. (C) Samples of 10 µg of each fraction were injected (i.d.) on the dorsum of mice followed by measuring of the hemorrhagic halo. (D) Samples with 0.1; 0.5; 1; 5 and 10 µg/mL SVMPs were incubated with platelet poor plasma. The reaction was initiated by addition of 0.025 M CaCl_2_ and the time to clot formation was measured.

Selected fractions were then subjected to a comparison of their functional activities under the same conditions. As shown in Figure 4, the proteolytic activity of each fraction on macromolecular substrates was variable: fraction Ic did not hydrolyze fibrin, and instead, it was efficient in the hydrolysis of gelatin. The opposite was observed with fractions IVa and IVb, which were fibrinolytic but not gelatinolytic. Fractions IIb and IIc hydrolyzed both, gelatin and fibrin (Figure 4A–B). These differences were also detected in the biological activities of SVMPs. As expected, only class P-III SVMPs (fractions Ic, IIb and IIc) were hemorrhagic with distinct potencies (Figure 4C). When tested for procoagulant activity, the fractions Ic, IIb and IIc reduced significantly the plasma recalcification time compared to controls. The procoagulant effect occurred in a dose dependent manner and each fraction presented different potencies with IIb and IIc being the most active fractions. Fractions IVa and IVb, corresponding to class P-I SVMPs, showed neither hemorrhagic nor procoagulant activities (Figure 4C-D). The detection of only enzymatic activities in fractions IVa and IVb suggests that they may be involved mostly on prey digestion.

**Figure 5:**
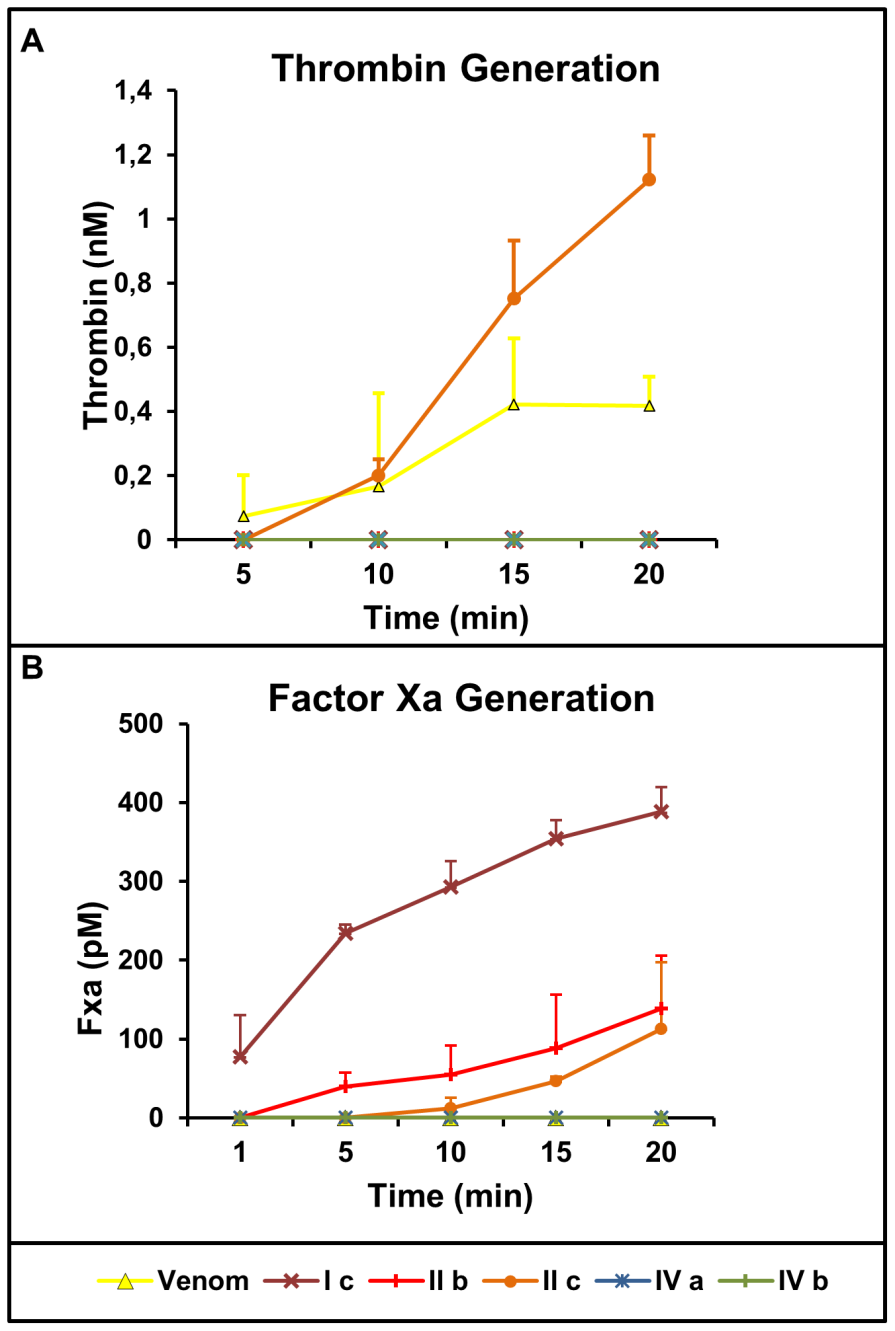
Prothrombin and Factor X activation by SVMPs fractions, as detected by S2238 and S2765 hydrolysis, respectively. Samples (1 µg/mL) of isolated SVMPs were incubated with 2 µM prothrombin (A) or 0.4 µM FX (B). Thrombin and FXa formed was determined after 1, 5, 10, 15 and 20 minutes of reaction. Assay conditions and quantification of thrombin and FXa are described in the Material and methods. Each point represents mean ±SD of three determinations.

SVMPs have already been described as activators of factor II and factor X independently of the presence of calcium [35]. Therefore, in order to understand the mechanism of their procoagulant activity, we tested the selected fractions in the activation of coagulation factors *in vitro*, thereby identifying the differences of SVMPs in the clotting process. Only fraction IIc was able to generate thrombin faster than the whole venom (Figure 5A) pointing out its direct effect in prothrombin activation (factor II). Regarding the factor X activation, only the fraction Ic was able to generate factor Xa *in vitro*, which was detectable from 5 minutes of incubation (Figure 5B). The fractions IIb and IIc showed some activity on factor X, although the data did not show statistical significance. These results confirm the action of fraction IIc in activation of prothrombin while fraction Ic acts at the direct activation of factor X.

**Figure 6:**
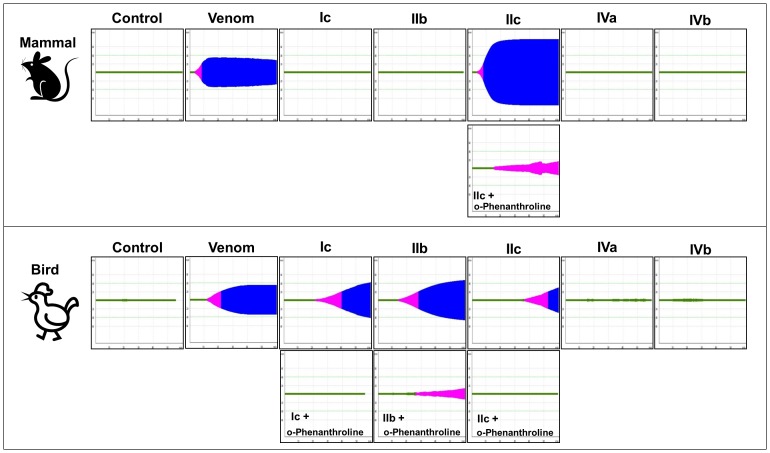
ROTEM profile of citrated rat or chicken whole blood samples treated with SVMPs fractions. Samples (40 µL) containing 10 µg SVMPs fractions were mixed with citrated rat or chicken whole blood samples (300 µL). ROTEM data are representative of three independent experiments and were acquired for 1 hour.

The next step was to verify if the different functional activities of SVMPs could play a role in their effectiveness to a broader spectrum of prey. For this purpose, we assessed the pro-coagulant activity of each fraction on citrated whole blood samples from rats and chickens, as representatives of mammalian and avian prey. Although the activation of coagulation factors is calcium dependent, most enzymes from viperid venoms can activate these factors even in the absence of divalent cations. Therefore we tested the coagulant activity of the fractions in the absence of calcium using rotational thromboelastometry. As shown in Figure 6, in the absence of calcium, the whole venom has a procoagulant action on both rat and chicken whole blood samples. The venom effect was faster on rat whole blood sample (green). Comparing the isolated fractions, only fraction IIc was effective in clotting rat whole blood sample, with the same clotting time (green) as the venom but resulting in a more stable clot (blue). On the other hand, fractions Ic, IIb and IIc were all able to clot chicken blood, but in a slower rate (green) than the whole venom. The most active on chicken blood was fraction IIb. Fractions IVa and IVb showed no activity on both systems, being similar to the PBS negative control. The fractions that showed pro-coagulant activity were treated with a metalloproteinase inhibitor (o-Phenanthroline) and after this treatment pro-coagulant activity was significantly inhibited in all fractions. Only a discrete and friable clot (pink) was observed in rat blood incubated with fraction IIc and in chicken blood incubated with fraction IIb after SVMP inhibition, confirming that metalloproteinases are the major enzymes responsible for the pro-coagulant activity presented in the fractions.

Table 2 summarizes the features of the SVMPs isolated in this study. Fraction Ic was identified as BnMPIIIc, a putative class P-III SVMP deduced from a cDNA sequence identified in the *B. neuwiedi* venom gland. Fraction Ic displayed high hemorrhagic activity and did not degrade fibrin. Fraction Ic was able to activate Factor X generating Factor Xa *in vitro.* In whole blood coagulation assays, fraction Ic was selective to clot chicken blood in the absence of calcium. Fraction IIb contained peptides identified in the class P-III SVMPs BnMPIIIc, jararhagin and bothropasin, from the venoms of *B. neuwiedi* and *B. jararaca*, respectively. Fraction IIb contained the usual characteristics of class P-III SVMPs with hemorrhagic, fibrinolytic and gelatinolytic activities and in whole blood assays, its action was observed only in chicken blood coagulation. Fraction IIc was also identified as a P-III class SVMP containing tryptic peptides identical to jararhagin or bothropasin but differed from the previous fractions by its remarkable prothrombin activation capacity and also by its higher clotting activity in rat blood samples compared to chicken. Except for a discrete fibrinolytic activity, fractions IVa and IVb, characterized as the class P-I SVMPs BnMPIb and BnMPIa, respectively, did not present any significant effect in the parameters related to the control of hemostasis evaluated in this study.

**Table 2:** Relative comparison of functional activities of isolated fractions.

			Biological Activities							
Samples	Identified SVMPs (Mass Spectrometry)	SVMP Class (SDS-PAGE)	Hemorrhagic	Fibrinolytic	Gelatinolytic	Coagulant	FII Activation	FX Activation	Mammals	Birds
**Venom**			+++	+++	+++	+++	++	-	++	++
**Ic**	BnMPIIIc	P-III	+++	-	++	++	-	+++	-	++
**IIb**	BnMPIIIc/	P-III	+++	++	+++	+++	-	+	-	++
	jararhagin/bothropasin									
**IIc**	jararhagin/bothropasin	P-III	++	+++	++	++	+++	+	+++	+
**IVa**	BnMPIb	P-I	-	++	-	-	-	-	-	-
**IVb**	BnMPIIa	P-I	-	+	-	-	-	-	-	-

The biological activities shown above were classified as low (+), moderate (++), or high (+++) for graphical representation.

## Discussion

There is increasing evidence that venom composition is significantly affected by post-genomic mechanisms (7) and that this plasticity is associated with diet [13], [36], [37]. One example is ontogenetic changes in venom that occur over the lifetimes of snakes in relation to changes in diet due to correlated changes in body size [38], [39]. In this regard, *Bothrops* snakes are mostly generalists that nonetheless show significant changes in diet over their lifetimes [38]. As such, to efficiently kill different prey, the toxin arsenal in these venoms must include toxins able to paralyze and kill different types of prey on which the snakes feed throughout their life cycle. To achieve such a toxin variety, a complex mechanism for modulation of gene expression has been described [6]. In venoms that disrupt hemostasis, the most variable toxin families (C-type lectins, serine proteinases and SVMPs) are encoded by multiple gene copies that undergo further selection and diversification at the post-transcriptional and post-translational levels [6]. As a result of this diversification process, it is hypothesized that toxins present in the snake venoms would act on distinct targets of coagulation and vascular systems of mammalian, avian, amphibian or invertebrate prey on which these animals feed. This assumption was first confirmed in this study in which the SVMPs isolated from *B. neuwiedi* venom were selective to different targets of the mammalian hemostatic system and acted with distinct potency on mammalian or bird blood.

Three distinct SVMPs P-III class were isolated and showed activity on different targets. Fractions Ic and IIc were unique in activating Factor X and Factor II of human coagulation system, respectively. Even the proteolysis profile of the SVMPs described in this study was different. Fraction Ic showed no action on fibrin but was effective in degrading gelatin, which is in agreement with its strong hemorrhagic activity associated with collagen digestion at the basement membrane of small vessels. Fraction IIb shows a broader spectrum of activities, with intermediate hemorrhagic, proteolytic and coagulant activity, but with no specific substrates. Acting together with the procoagulant serine proteinases, these enzymes would induce a rapid consumption of blood components and damage in blood vessels that would impair prey mobility and survival.

In parallel to the selectivity to different targets, we showed that SVMPs act distinctly on the coagulation system of different animals. Our initial tests were performed using the human coagulation system that is well adapted to the study of snake toxins. Further, we also tested the effects of isolated fractions on the coagulation of rat and chicken blood, which are more similar physiologically to snake prey. By using the thromboelastometric assay, only fraction IIc was effective in coagulation of the rat blood while fractions Ic and IIb were capable of clotting chicken blood. According to Martins et al. [38], *Bothrops* species are usually diet generalists, but the authors highlight that most subspecies of *B. neuwiedi* complex are mammal specialists. In this case, birds could represent an important predator of *B. neuwiedi* snakes explaining why the venom showed even more effectiveness against avian blood coagulation system. Thus, a dietary pattern between the species of *Bothrops* snakes is not likely to be the only factor modulating plasticity and venom composition could be affected also by other ecological parameters acting on the expression of different venom components.

The presence of isoforms of SVMPs and other venom proteins is a consequence of the evolutionary process of those protein families in snakes favoring diversification of their venom components. Besides the high mutation rates that follow gene duplication processes [4], [5], [40], alternative transcription [41] and recombination between the genes and primary mRNA transcripts have been demonstrated [29] for genes of snake venom serine proteinases and SVMPs, respectively. These mechanisms result in the production of structurally similar molecules in which the essential motifs, as catalytic sites, are conserved. These variable regions are often located in more flexible sites that may be involved in substrate binding and, therefore, driving the enzyme selectivity to distinct targets. The sequence variability in molecules with high structural similarity implies in several experimental problems encountered during this study. The complete purification of the different toxins belonging to the same class of SVMPs was impossible in some cases. For this reason, we decided to carry on the study using fractions with predominance of SVMPs and devoid of serine proteinases. Other problem recurrent in the study concerned the precise characterization of the isolated bands by mass spectrometry. Some internal peptides are conserved in several members of the same SVMP class and are easily detected by mass spectrometry. Therefore, the identification of protein bands shown in Table 1 is only an indication of their SVMP nature. In this regard, the complete characterization of primary structures would be an essential requirement for depicting the structure/function relationships that would completely enlighten this first evidence of functional variability within the same toxin family.

Comparing the isolated *B. neuwiedi* SVMPs to already described toxins, two fractions containing class P-I SVMPs, identified in this study as BnMPIa and BnMPIb, showed only proteolytic action on fibrin and no activity on blood clotting. In previous studies, Modesto et al. [42] demonstrated that insularinase, a P-I class enzyme from the venom of *B. insularis*, which shows sequence identity with BnMPIa and BnMPIb higher than 90%, has pro-coagulant activity by activating coagulation Factor II, suggesting that targets and general function may differ even in closely related structures. Similarly, fraction IIc was identified in this study as jararhagin/bothropasin according to sequence of internal peptides identified by mass spectrometry. Jararhagin [43] and bothropasin [44] are isoforms of class P-III SVMPs from *B. jararaca* venom with sequence identity higher than 95% and only approximately 65% identity with BnMPIIb and BnMPIIc, indicated as a possible identification by mass spectrometry. Interestingly, neither jararhagin nor bothropasin show activation of human coagulant factors.

In contrast to the fractions containing P-III class SVMPs, those containing P-I class SVMPs showed little effects on the targets of hemostatic system accessed in this study. They presented only proteolytic activity on fibrin, which is consistent to previous data on the literature [34]. Thus, it is reasonable to expect that the major SVMP involved in prey killing or immobilization are those of the P-III class. In this scenario, P-I class SVMPs are more likely involved in the digestive function of snake venoms. Nevertheless, it is important to point out that a digestive activity of prey tissues may have a counterpart in the very deleterious effects of P-I class SVMPs in local lesions evoked in human victims of snake bite. Indeed, a strong pro-inflammatory action has already been reported for BaP1, a class P-I SVMP from *B. asper* venom [45], [46] and neuwiedase, a P-I class SVMP from *B. neuwiedi* venom [47].

At this point, it is important to emphasize the relevance of the functional diversity of SVMPs for human victims of snake bites. Together with the digestive function of P-I class SVMPs, P-III class enzymes act in different targets of coagulation and fibrinolysis systems, endothelial cells and extracellular matrix components, accounting for most of systemic and local effects of snake bites. Fortunately, SVMPs are the predominant antigens recognized by commercial antivenoms [27]. However, it still has to be proven if the antibodies present in commercial antivenoms are able to recognize the distinct forms of SVMPs present in the venoms of *Bothrops* snakes and if they are able to neutralize the interaction of these SVMPs with their different targets. Therefore, understanding the diversity of this class of toxins is still very important to design antivenoms able to neutralize toxins responsible for the death and injury resulting from snake bites in humans.

## Concluding Remarks

Many *Bothrops* snakes in South America have a paedomorphic venom in which the predominant phenotype is the abundance of P-III class SVMPs in venoms throughout their life cycles [48], [49]. A striking feature of these enzymes is the versatility they show towards different biological systems [24], [50] and this functional diversity may be due to their evolutionary origin in targeting different prey. The functional diversity of SVMPs shown in this study clarify the importance of this class of toxins for the feeding ecology of *Bothrops* snakes and suggest further directions for studies on the design of more efficient antivenoms.
